# ‘Borono-lectin’ based engineering as a versatile platform for biomedical applications

**DOI:** 10.1080/14686996.2017.1411143

**Published:** 2017-12-19

**Authors:** Akira Matsumoto, Yuji Miyahara

**Affiliations:** ^a^ Institute of Biomaterials and Bioengineering, Tokyo Medical and Dental University, Tokyo, Japan; ^b^ Kanagawa Institute of Industrial Science and Technology (KISTEC-KAST), Kawasaki, Japan

**Keywords:** Boronic acids, borono-lectins, drug delivery systems, sialic acids, diabetes, insulin, siRNA, 30 Bio-inspired and biomedical materials, 212 Surface and interfaces

## Abstract

Boronic acids are well known for their ability to reversibly interact with the diol groups, a common motif of biomolecules including sugars and ribose. Due to their ability to interact with carbohydrates, they can be regarded as synthetic mimics of lectins, termed ‘borono-lectins’. The borono-lectins can be tailored to elicit a broad profile of binding strength and specificity. This special property has been translated into many creative biomedical applications in a way interactive with biology. This review provides a brief overview of recent efforts of polymeric materials-based engineering taking advantage of such virtue of ‘borono-lectins’ chemistry, related to the field of biomaterials and drug delivery applications.

## Graphical abstract

**Figure:**
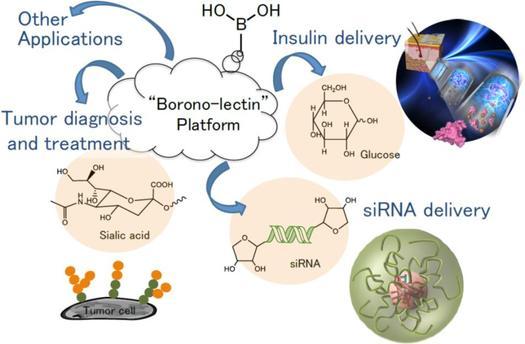

## 1. Introduction

**Scheme 1.**
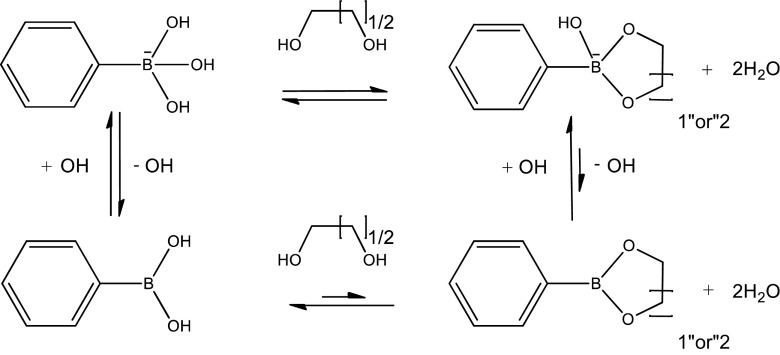
Reversible boronate ester formation in aqueous solution.

Boronic acid (BA) derivatives can readily interact with *cis*-diols, a commonly found motif in biomolecules including sugars [1–4] and ribose [5–10], through reversible boronate ester formation in an aqueous solution (Scheme 1). For its carbohydrate-binding capability, BA can be regarded as a synthetic mimic of lectins (carbohydrate-binding proteins), termed ‘borono-lectin’ [11–15]. BA is also known for its vital roles in homeostasis of plants and arguably even in the origin of life; recent studies have described its effect to optimally stabilize RNA through interaction with the ribose functionality as proposed mechanism for the ‘RNA world’ persistence under prebiotic conditions [16–18]. These and the fact that a variety of BA chemistry today prevails as chemotherapeutics and other remedies [19–21] may support inherent compatibility of these compounds with life. The strength of interaction observed for monomeric state BA is essentially weak as compared to proteins, e.g. the binding stability of BA-sugar interactions is 3 to 4 orders of magnitude smaller than those typically observed for lectins. Furthermore, furnishing BA with an ability to distinguish glycoconjugate-scale structural complexity, which is commonly present with natural lectins [22], is quite challenging unless it is carefully engineered [23]. Nonetheless, such ‘weak’ and ‘vague’ interactions can be dramatically improved once they are polymerized taking advantage of the polyvalent effect, making itself a unique class of bio-application platform [24–30]. That is to say, such dynamic interaction can be exploited to address otherwise difficult challenges such as continuous monitoring, environment-sensitive and bio-interactive applications, in which temporal, reversible or oscillatory patterns of biomolecules are matter of interest. One can also tailor the ‘borono-lectin’ to elicit a divergent profile of binding strength and specificity on the basis of stereochemistry and controlled electronic effect [31,32]. Moreover, thanks to recent advancement of Suzuki-Miyaura coupling chemistry [33], there is an ever-increasing lineup of BA derivatives that are accessible for reasonable price. Besides the versatility, also noteworthy is that some groups of BA can undergo a sharp inversion in the state of hydration in synchronization with the molecular recognition; typically, being hydrophilic when charged in the presence of the binding targets and *vice versa*. This feature, especially when combined with amphiphilic type of polymeric backbone, further widen the utility of BA in materials engineering, as it leads to many creative principles for fine-tuning or switching the hydration and more complex molecular assemblies in a way interactive with biology. With a special focus on these unique features of BA, among others, herein we aim to provide a brief summary of recent efforts of materials engineering, including our own contributions, that are relevant to the field of biomaterials and drug delivery applications.

### 2.1. Intracellular environment-selective delivery of siRNA

We commence this review by mentioning to an example of our own contributions that we think best illustrates the virtue of the BA chemistry, taking full advantage of its unique properties in combination with polymeric materials.

**Figure 1.**
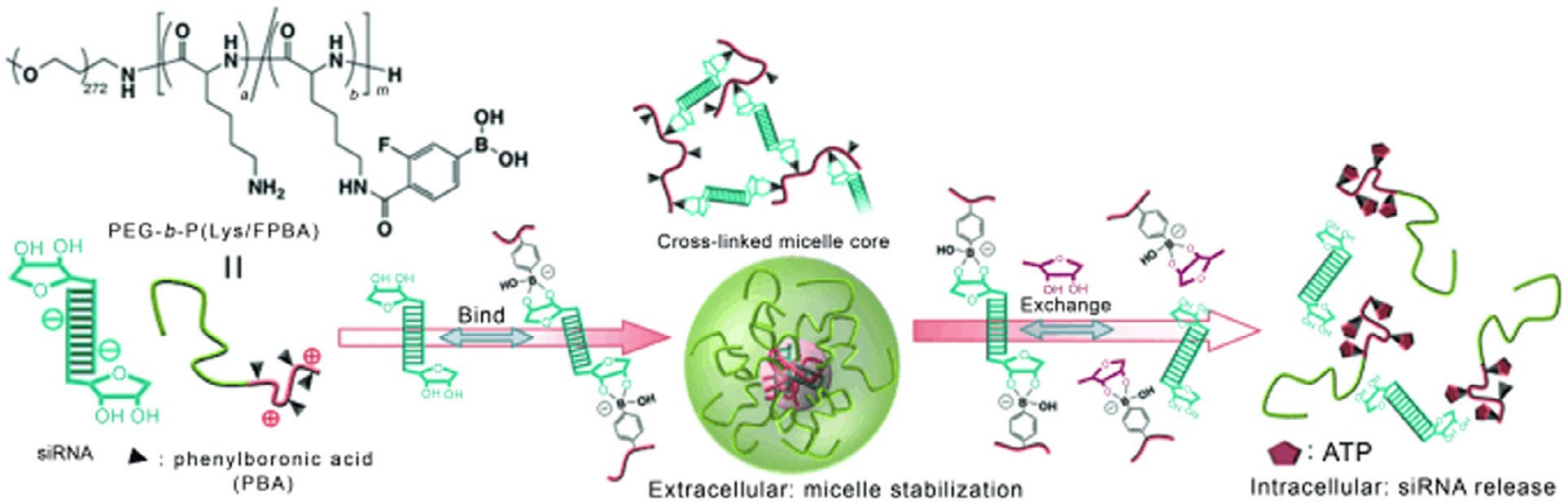
Schematic representation of the phenylboronic acid-based strategy for siRNA delivery; the chemical formula of the polymer, enhanced stability of the micelle, and the mechanism of selective intracellular release are shown. Reprinted from Ref. [47] with permission. © 2012, WILEY-VCH Verlag.

There is a growing interest in the delivery of small interfering RNA (siRNA) for its ability of gene silencing in a highly sequence-specific fashion [34,35]. One major approach is a formulation into polyion complex (PIC) micelles that instantly form in an aqueous environment, through electrostatic interactions between anionic siRNA and cationic polymers [36]. The greatest challenge is optimally stabilizing the PIC-micelle; while in the bloodstream it must be robust enough to protect siRNA from the endogenous RNase attack, however, once reaching the site of intracellular targets it is required to adversely destabilize so to release siRNA. To meet these criteria, many attempts have been made, a majority of which focus on either one or combinations of the following three methodologies, namely, covalent conjugation of siRNA to the homing polymer [37–41], introduction of hydrophobic moieties to reinforce the core-aggregate [42–44] and cross-linking the core aggregate by the disulfide bridging [45,46]. However, these combinatorial approaches inevitably yield a complexity in structure and method of preparation. We have demonstrated that BA-ribose interaction, which has been long-studied as ligand chemistry in chromatography, indeed provides a sophisticated solution (Figure 1). Our strategy capitalizes solely on the phenylboronic acid (PBA) functionality, which incorporates all the aforementioned three modes of stabilization effects (Figure 5) [47]. Our platform cationic polymer was poly(ethylene glycol)-*block*-poly(L-lysine) (PEG-*b*-PLys), the lysine residues of which were functionalized with 3-fluoro-4-carboxyphenylboronic acid (FPBA) to graded degrees. In the first stabilization mode, the polymer pendent PBA can serve as group for chemical conjugation with ribose of siRNA. Then, upon electrostatic condensation to form the PIC-micelle, intermolecular cross-links prevail due to bis-bidentate ribose arrangement at each 3′ end of the double-stranded siRNA thereby further stabilizing the complex (2nd mode). Furthermore, PBA is unique in that it undergoes a dramatic inversion in the state of hydrophobicity depending on the degree of acid disassociation, which is sensitive to the ribose or other competing moieties in the milieu, providing an additional (3rd) mode of reversible stabilization.

**Figure 2.**
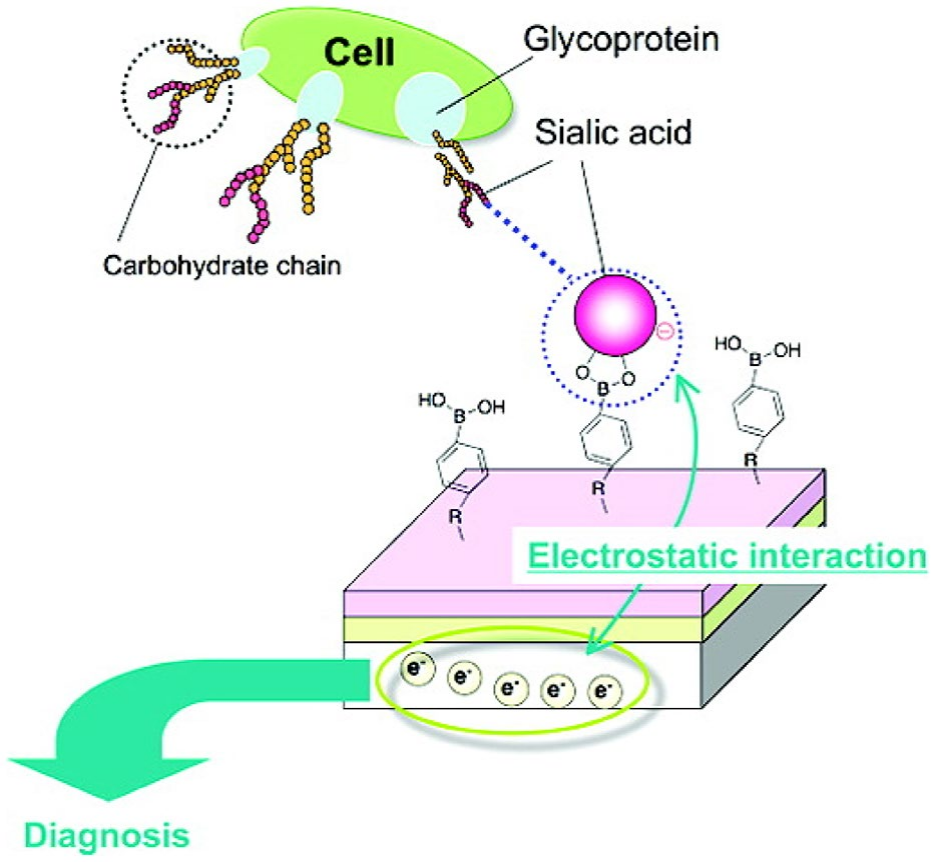
Potentiometric SA detection at cell membrane using PBA modified electrode. Reprinted from Ref. [60] with permission. © 2009, American Chemical Society.

**Figure 3.**
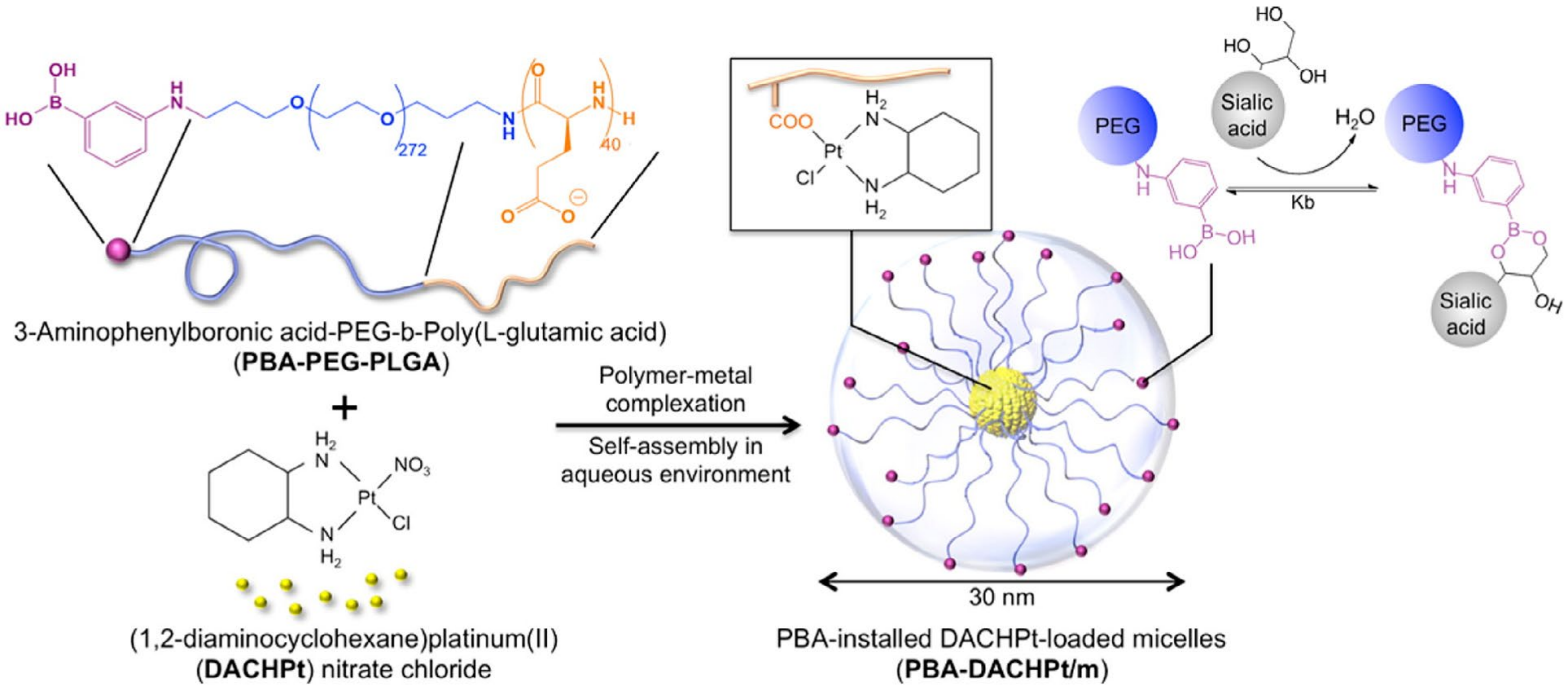
Preparation of PBA-installed DACHPt-loaded micelles by self-assembly through polymer–metal complex formation between DACHPt and PBA-poly(ethylene glycol)-*b*-poly(l-glutamic acid) in distilled water. PBA moieties on the surface of the micelles can bind to SA. Reprinted from Ref. [64] with permission. © 2013, American Chemical Society.

**Figure 4.**
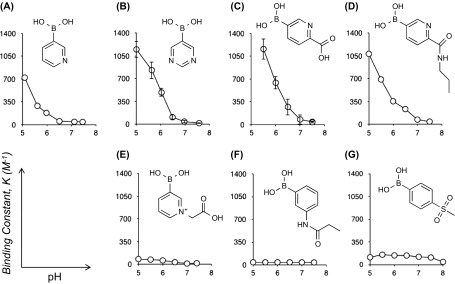
Binding constants (*K*, M^−1^) for SA binding to a variety of boronic acids as a function of pH, as determined by B^11^NMR analysis; A: 3-pyridylboronic acid, B: 5-pyrimidine boronic acid, C: 5-boronopicolinic acid, D: (6-propylcarbamoyl)pyridine-3-)boronic acid, E: 3-borono-1-(carboxymethyl)pyridine, F: 3-propionamidophenylboronic acid, G: 4-(methylsulfonyl)benzeneboronic acid. Values in B and C represent mean S. D. (n = 3). Reprinted from Ref. [74] with permission. © 2012, Royal Society of Chemistry.

**Figure 5.**
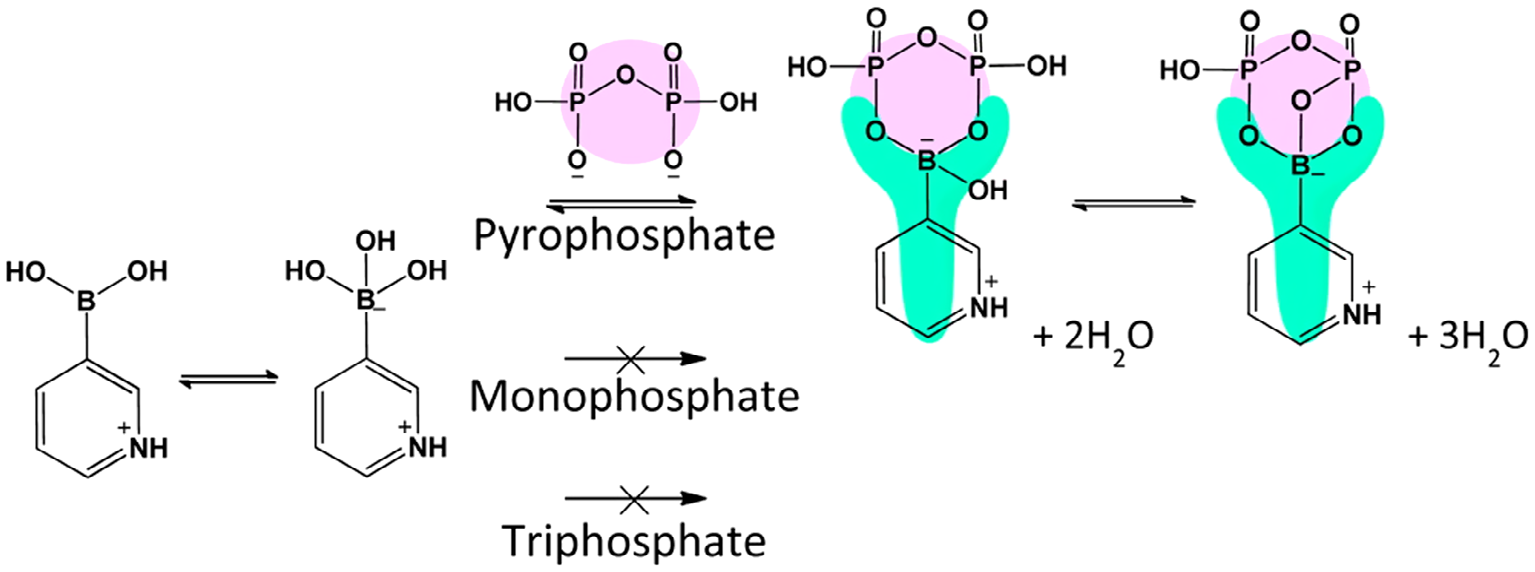
Diphosphate-specific recognition under weakly acidic pH conditions identified with by some boronates with relatively strong acidity, as determined by ^11^B and ^31^P NMR studies. Reprinted from Ref. [75] with permission. © 2015, American Chemical Society.

It was demonstrated that the specific binding between the pendent PBA and 3′end ribose at both ends of the double stranded siRNA, along with the hydrophobic interaction of PBA, cooperatively contribute to the stability of the complex in quasi-extracellular conditions. The complex could be optimized to cause disruption in response to adenosine triphosphate (ATP) only when the concentration exceeds that of intracellular environment, while remaining insensitive to any other competing sugars. As a result, the optimized complex exhibited a dose-dependent silencing capability of the polo-like kinase 1 (PLK-1) gene, a well-known proto-oncogene in the human renal carcinoma cell (OSRC-2) line, with no appreciable cytotoxicity [47]. Such PBA-assisted PIC-micelle may have potential for intracellular environment-selective delivery of siRNA and other small RNAs.

A similar effort by Engbersen et al. which is based on multifunctional poly(amido amine) incorporating the pendent PBA, aiming to achieve environmentally sensitive and safer gene delivery systems, has also been reported [48]. Consistent with the above-described system, the PBA functionality aided in stabilization of self-assembled nanoparticles and DNA- and siRNA-polyplexes formed at physiological pH, due to reversible cross-linking between the PBA and neighboring Lewis bases. The competing sugar moieties such as D-sorbitol and dextran could modulate the stability of the polyplexes and the interaction with glycoproteins on the surface of the cells, both representing a determinant for the resultant transfection and silencing efficiencies.

The ATP-responsive BA chemistry has also been studied as a mode to modulate enzymatic activity in a recent report by Aida et al. [49]. Water-soluble linear polymers bearing multiple guanidinium ions (Gu^+^) and BA pendants were synthesized as ATP-responsive modulators for enzyme activity. These polymers (GumBAn polymer) strongly bind to the phosphate ions (PO^4–^) present on proteins and 1,2-diol units of ATP via the Gu^+^ and BA pendants, respectively. It was demonstrated that trypsin (Trp) can be deactivated by hybridization with GumBAn. However, upon addition of ATP, Trp was liberated to retrieve its hydrolytic activity due to a higher affinity to ATP than Trp, leading to an ATP-responsive mode of the cell detachment. Importantly, the event was manifested at quite a low range (1–10 μM) of [ATP] which is relevant to the physiology.

### 2.2. Sialic acid interaction-based applications and some new aspect of molecular recognition

Glycosylations, or the alternations of the glycoforms, are dynamic and stage-specific processes related to both normal and pathological events including development, differentiation, infection, genetic disorders and cancers [50–54]. However, the precise roles of glycans remain to be completely elucidated largely due to its tremendous diversity in structure. Sialic acids (SA or N-acetylneuraminic acid) is a family of sugars that constitutes a significant proportion of glycan structures. Given their outermost arrangement and uniquely anionic polarity, it mediates a variety of physiological and pathological cell processes, and thus can provide an accessible ‘code’ to tell the state of glycans in the context of cellular events. For example, sialylation is typically altered in cancers; increased expression of sialylated glycans is a common manifestation of cancer progression, poor prognosis and higher metastatic potential [55–57]. Therefore, determination of the glycan SA is relevant to diagnosis of these cancerous conditions. Furthermore, SA-specific molecular recognition leads to capability of targeting therapeutic agents to highly sialylated epitopes or tumor cells. BA-related chemistry does provide solutions for these challenges. A capability of BA of specifically binding with SA among other glycan-related sugars had been clarified based on nuclear magnetic resonance (NMR) studies; the origin of the SA-specificity has been attributed to multiple metastable binding sites in the complex along with intramolecular stabilization effect involving B-N or B-O interactions [58,59]. Taking advantage of this finding, we have reported a BA-based potentiometric detection technique for SA as a new platform for noninvasive and label-free cytology [60,61]. A PBA modified self-assembled monolayer was immobilized on the surface of a gold electrode, which was then used as an extended gate of a field effect transistor (FET). The specific binding between negatively charged SA and PBA led to a change in the surface potential of the FET. The resultant *in situ* chemical-to-electrical signal-transduction mode proved an efficient means to quantify the cell-surface expressed SA on a label-free and real-time format (Figure 2). It was able to distinguish the SA alternations on the surface of erythrocytes [56] as well as metastatic murine melanoma cells (B16-F10) [57], simply by placing the known-count living cell suspensions onto the electrode, each relevant to the diagnosis of diabetes and tumor metastatic potential.

The PBA-SA interaction described above has also been exploited as a means to label sialylated epitopes *in vitro* and *in vivo* [59,62]. For example, Crich et al. have reported a gadolinium macrocyclic complex conjugated with PBA (Gd^III^-dota-en-pba) as a SA-specific contrast agent for *in vivo* magnetic resonance imaging (MRI) [63]. This conjugate proved feasible for the SA-enhanced tumor imaging on mice model bearing tumor xenograft obtained by subcutaneous injection of B16-F10 cells.

We have pioneered an approach of a PBA-modified (as a ligand to SA) polymeric micelle encapsulating anti-cancer drugs as a route to obtain tumor-specific chemotherapy (Figure 3) [64]. Ligand-mediated drug targeting is an attractive strategy for increasing the efficiency of chemotherapies. Indeed, SA has been targeted *in vitro* by using lectin [65] and antibodies [66], such as the tumoral marker CA19–9, for the detection of Sialyl-Lewis^a^ in gastrointestinal tumors. Nevertheless, these approaches have been difficult to translate *in vivo*, largely due to their immunogenicity. Moreover, since SA is universally present on biological surfaces including red blood cells and the luminal surfaces of vascular endothelium [66–68], the ligand is required to ‘temporarily silence’ until reaching the site of tumors. In this context, a synthetic ligand system using BA can offer an intriguing solution to avoid the immunogenic issue while also achieving the pH-sensitive action specific to tumor microenvironment. That is to say, BA ligand can be modulated so that it is shielded by other competing sugars such as glucose while in the bloodstream (pH 7.4), and become increasingly SA-specific only when exposed to acidic conditions implicated in intratumoral environment; the local pH of hypoxic tumoral microenvironment is commonly lower than the surrounding tissues, typically in the range of 6.5 in comparison with that for healthy tissues (i.e. 7.4), due to disturbances in the metabolic balance of neoplastic cells [69–71]. The micelle was prepared through coordination between an anticancer agent, (1**,**2-diaminocyclohexane)platinum(II) (DACHPt), and the carboxylic groups of PBA end-functionalized poly(ethylene glycol)-*b*-poly(L-glutamic acid) [PBA-PEG-*b*-PLGA] copolymers in an aqueous solution [64]. The ability of the PBA-modified micelle to bind with SA epitopes in cancer cells was first validated *in vitro* by evaluating the cellular uptake and cytotoxicity against B16F10 cells. The significant effect of the BA ligand to enhance antitumor activity was also confirmed *in vivo* for both murine orthotopic and metastatic tumor models. Most importantly, these enhancements in antitumor activity did not come at the expense of side effects; the body weight of the mice remained stable even after the repeated administration of the micelles. Inspired by these findings, many related research efforts of diagnostic [72,73], and therapeutic applications are ongoing.

Quite recently we have found that a group of heterocyclic boronic acids, based on the pyridine structures, can serve as remarkably strong and SA-selective binders, producing the binding constants orders of magnitude higher than those previously reported (Figure 4) [74]. Note that the binding constant between SA and meta-amide substituted phenylboronic acid, a gold-standard structure which has proven to be an efficient SA-binder for the above-described therapeutic and diagnostic applications, was at most 40 M^−1^ [54], whereas some derivatives we identified revealed values well exceeding 1000 M^−1^, to our knowledge the highest levels ever reported. Remarkably, these interactions strengthen under weakly acidic pH conditions associated with hypoxic tumoral microenvironment, resulting in a highly SA-selective interaction among the other common sugars present in biological samples. In particular, 5-boronopicolinic acid was found suitable for further chemical conjugation with well-preserved SA-binding capability. Based on this structure, a fluorescence-labeled derivative was prepared and tested *in vitro* for lectin-competitive binding assay as well as glycan array assays. These assays collectively evidenced its capability of binding with biologically relevant sialylated glycoconjugates, beyond the monosaccharide level recognition, accompanied by a marked SA-specificity. Also, worth mentioning is that an excellent water solubility is generally found with these derivatives, which is otherwise (e.g. phenylboronic acid) often an issue limiting the range of medical applications. Taken together, these findings should offer an attractive alternative to a number of ongoing BA chemistries aiming to achieve tumor-specific chemotherapies and diagnoses.

Interestingly, we recently found that some of those heterocyclic boronic acids mentioned above can also undergo pyrophosphate (PPi)-specific binding in a pool containing other phosphates or organophosphorus compounds (Figure 5) [75]. PPi is involved in many important cellular enzymatic reactions. For example, during DNA replication reaction catalyzed by DNA polymerase, it is stoichiometrically produced on each occasion of the single base synthesis. Therefore, the detection of PPi is relevant to DNA sequencing, a technique known as pyrosequencing [76,77]. Furthermore, a change in fluid PPi concentration has been implicated in several pathological conditions including tumors [78,79]. Aside from the gold-standard enzymatic determination methods, there are some reported synthetic PPi sensors capitalizing on binuclear metal coordination chemistry in which each metallic center is designed to chelate with two PPi oxygen atoms thereby inducing changes in optical and electrical properties of the complex [80]. However, these interactions are in practice irreversible; the binding constants (K) found with these interactions are typically on the order of 10^8^ M^−1^, whereas those between heterocyclic BA and PPi are on the order of 102–103 M^−1^. As a result, those chelator type sensor molecules may not be suitable for continuous monitoring and other potential biomedical applications, where more dynamic interactions prevail with biological significance. In this regard, BA compounds may find unique applications.

### 2.3. Toward smart therapy of diabetes

**Figure 6.**
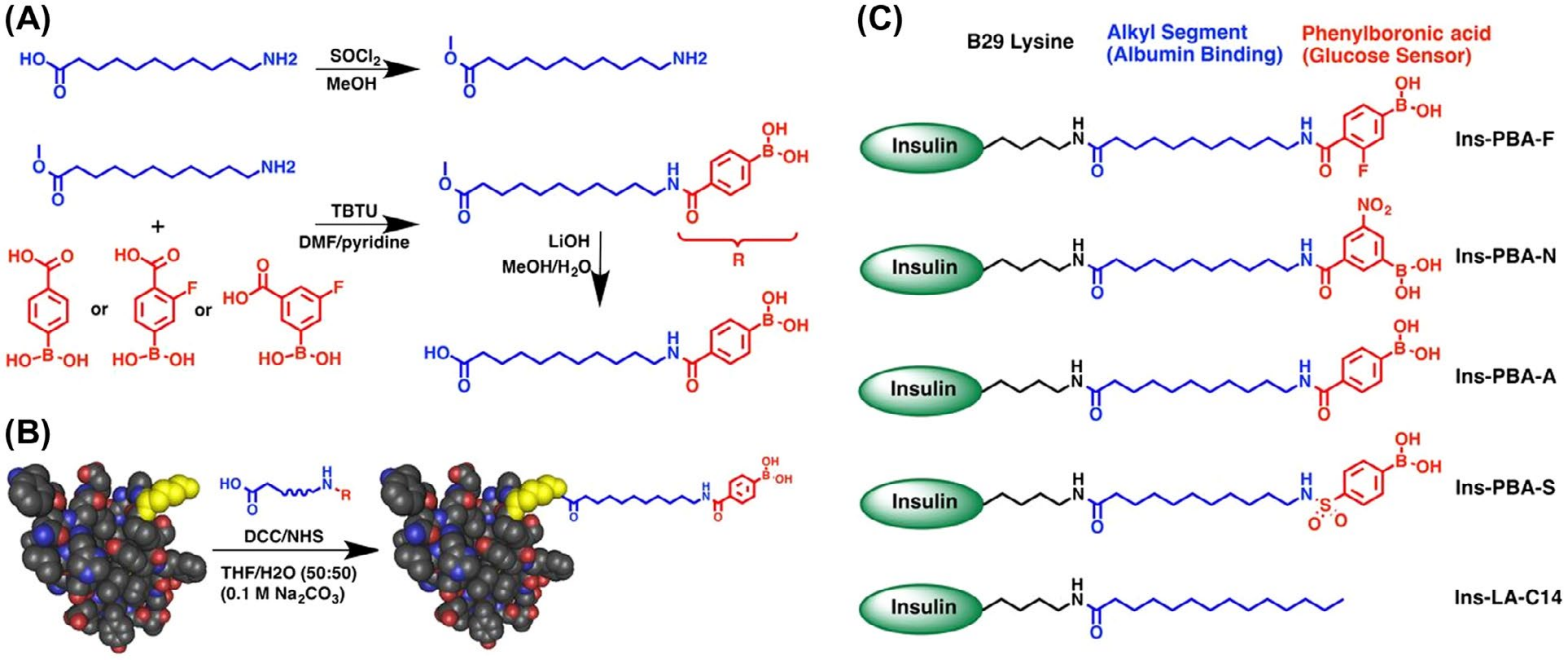
Schematic illustrating the strategy for insulin modification. (*A*) Generalized scheme to prepare small molecules used in the preparation of Ins-PBA-F, Ins-PBA-N, and Ins-PBA-A. (*B*) Final carboxylic acid-containing small molecule is conjugated to the native human insulin protein through the ε-amine of the lysine at the B29 position (yellow). (*C*) Structures of the four PBA-modified long-acting insulin derivatives along with the positive control, Ins-LA-C14, commercially known as insulin detemir. Reprinted from Ref. [90] with permission. © 2015, PNAS.

Among a growing lineup of medications for diabetes, insulin therapy continues to be a primary option in clinical practice for both palliative and preventive purposes [81–86]. Currently, the most common modality of this treatment is the patients’ self-administration, termed ‘open-loop’ insulin delivery. However, this method inevitably suffers from inaccuracy of the dose control, where the overdose must be strictly avoided otherwise causing acute and fatal hypoglycemia. There have been constant efforts to develop insulin variants with tunable pharmacokinetics [87,88]. Above all, long-acting variants, as opposed to native unmodified or fast-acting types, may offer great benefits in daily scenes of the management by minimizing the frequency of administration [89]. However, this type of insulin lacks the ability of acute (spike-like) response. Anderson et al. have recently developed a new type of ‘smart’ long-lasting insulin that is responsive to glucose, based on a creative BA involved engineering (Figure 6) [90]. A series of aliphatic molecules containing PBA moieties were synthesized for covalent conjugation to insulin. The use of an aliphatic domain was inspired by the design of (clinically used) long-acting insulin detemir to afford binding to serum albumin, or other hydrophobic components in serum, for prolonged circulation half-life. The incorporation of PBA was intended for a glucose-sensing element within the conjugate. The best-performing derivative (Ins-PBA-F: Figure 6) was able to rapidly ameliorate blood glucose in a diabetic mouse model following glucose challenge, providing the glycemic control that is superior even to native insulin. Also, remarkably, administration of this derivative led to a reduction in hypoglycemic index in healthy mice, compared even to those treated with native insulin. A detailed glucose-responsive mechanism and the safety issue of using such covalently modified insulin remain to be elucidated. Even so, in combination with other well-established interfacing technologies including insulin pumps, infusion devices or controlled release materials, this approach should offer an attractive adjunct to the current therapeutic technologies.

**Figure 7.**
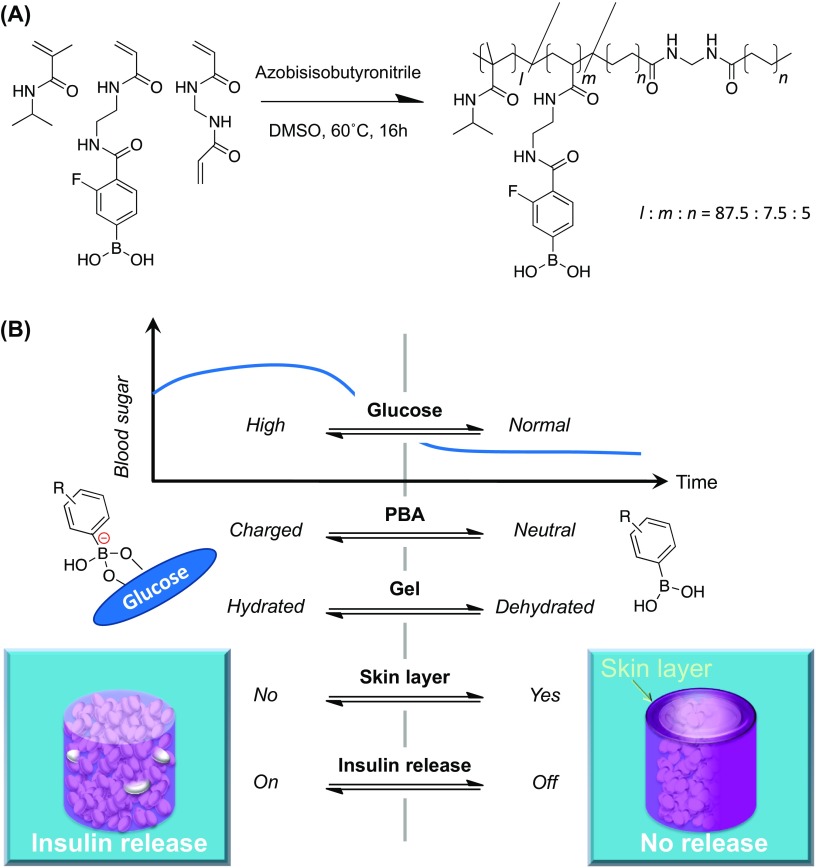
(a) Chemical structure of glucose-responsive gel: monomers and their polymerized (cross-linked) chemical structures shown with their optimized molar fraction (*l:m:n* = 87.5:7.5:5) to yield the glucose-sensitivity under physiological conditions (pH 7.4 and 37 °C) accompanied by a threshold concentration of glucose at normoglycemic 100 mg/dl (above which the gel delivers insulin). (b) Schematic illustration of ‘pancreas-like’ self-regulated insulin delivery function of the gel. Reprinted from Ref. [102] with permission. © 2017, American Chemical Society.

To take one step further, we pursue the development of a synthetic polymer gel-based insulin delivery system capitalizing on PBA chemistry, which is applicable to any types of insulin including the above. The development of self-regulated insulin delivery systems has been a constant topic of biomedical engineering. To this end, the use of glucose oxidase (GOD) and sugar-binding lectin (Concanavalin A) are two major approaches to endow the homing polymer materials with the glucose-sensitivities [91–93]. However, these protein-based materials are intolerant of long-term use and storage due to their denaturing and cytotoxic natures. Consequently, no successful clinical translation of these systems has hitherto been made. Therefore, some researchers envision to provide a PBA gel-based and thus totally synthetic alternative [94,95]. Our previous studies had demonstrated that a glucose-dependent shift in the equilibria of PBA, when integrated with optimally amphiphilic acrylamide gel backbone, could induce a reversible, glucose-dependent change in hydration of the gel [96]. The resultant abrupt and rapid change in hydration of the gel, under optimized conditions, led to formation of a gel-surface-emerging, microscopically dehydrated layer, so-called ‘skin layer’, providing a mode that is able to effectively switch the release (diffusion) of the gel-loaded insulin (Figure 7) [96,97]. We have also reported that the chemical structure of the gel could be optimized so that it undergoes the above-mentioned performance under physiologically relevant conditions, accompanied by a remarkably gated manner response to the level of normoglycemia [98–101]. This system is free from all electronics that are of absolute necessity for the current ‘closed-loop’ artificial pancreas, i.e. sensors, battery, motors, microcomputers, algorithm and electrical or wireless communication modules. In other words, all these functions have been molecularly programmed in the gel. The pre-gel solution, which instantly gels upon heating, can fill virtually any desired shape and dimension, compatible with other existing medical device structures such as needles and catheters. To validate such idea, our most recent study has described the gel-combined device confined within a single catheter, which exhibits an artificial pancreas–like function *in vivo* [102]. Subcutaneous implantation of the device in healthy and diabetic mice establishes a closed-loop system composed of ‘continuous glucose sensing’ and ‘skin layer’–regulated insulin release. Consequently, the glucose metabolism was controlled in response to interstitial glucose fluctuation under both insulin-deficient and insulin-resistant conditions with at least 3-week durability. Our ‘smart gel’ technology could offer a user-friendly and remarkably economic (disposable) alternative to the current state of the art, thereby facilitating availability of effective insulin treatment not only to diabetic patients in developing countries but also to those patients who otherwise may not be strongly motivated, such as the elderly, infants, and patients in need of nursing care. Our ongoing efforts have been directed toward these possibilities for clinical translation.

### 2.4. Iminoboronate chemistry as a new mode of bioactive applications

**Figure 8.**
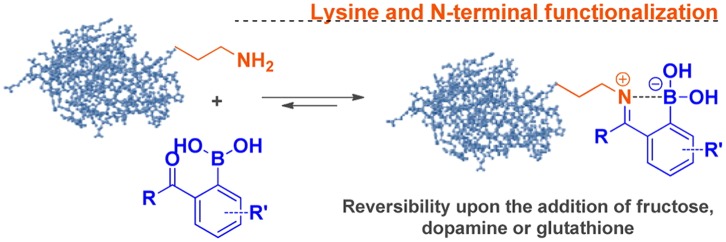
Lysine ε-amino group modification based on the formation of stable imines with 2-formylbenzeneboronic acid. Reprinted from Ref. [102] with permission. © 2012, American Chemical Society.

Gois et al. have recently reported that PBA derivatives with either 2-formyl- or 2-acethyl substituent groups are able to form a remarkably stable iminoboronate when reacting with primary amines in an aqueous environment (Figure 8) [103,104]. The stabilization of the imine, which is otherwise readily susceptible to hydrolysis, was reasoned by a dative bond between the imine nitrogen and the neighboring boronic acid (B-N interaction), which can be induced and robustly tolerate under physiological aqueous conditions. Importantly, this iminoboronate linkage can be readily cleaved by acidification and in the presence of competing diols or some reducing agents, proposing a new method for reversible protein modification specific to the lysine’s ε-amino group and N-terminal.

**Figure 9.**
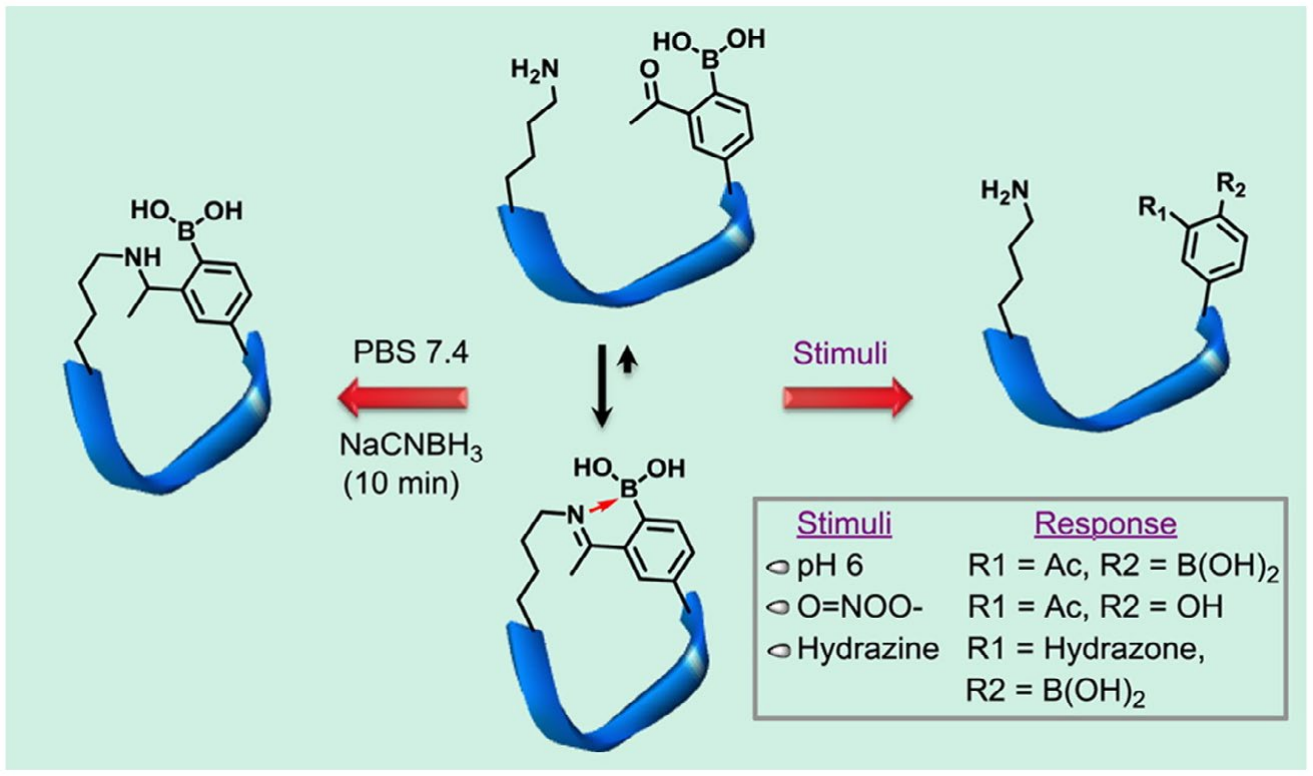
Iminoboronate-mediated peptide cyclization in which intramolecular iminoboronate formation allows spontaneous cyclization under physiologic conditions to yield monocyclic and bicyclic peptides. Importantly the iminoboronate-based cyclization can be rapidly reversed in response to multiple stimuli, including pH, oxidation, and small molecules. PBS; phosphate buffered saline. Reprinted from Ref. [105] with permission. © 2016, American Chemical Society.

Bandyopadhyay and Gao have made use of this chemistry as a means to accomplish peptide cyclization [105]. There is a growing interest in the synthesis of cyclic peptides mimicking the structure and function of their natural counterparts. Among a number of the peptide cyclization strategies, the disulfide chemistry represents so far the only non-permanent or reversible type of linkages enabling the design of ‘smart’ peptides that can turn on or off their activity in response to biological stimuli. Gao et al. demonstrated that iminoboronate linkages could also play this role. In particular, a series of RGD (an integrin-recognition motif) containing sequences bearing artificial boronate-functionalized amino acid moieties in a way flanking the above were designed and optimized so as to spontaneously yield mono- or bicyclizations via intramolecular iminoboronate formation (Figure 9). The iminoboronate linkages incorporated into these peptides were reversibly cleavable in response to acidification, oxidation, and addition of some exogenous small molecule modulators. Furthermore, fluorescence-labeled version of the iminoboronate-cyclized RGD peptides were tested for the binding with SKOV3 cells, an ovarian cancer cell line known to overexpress the αvβ3 integrin. As expected, the fluorescence staining of the cells was effectively switched when altering the pH between 7.4 and 6.0, under which the peptide undergoes conformational change between cyclized and linearized states.

Gao et al. have further described the iminoboronate chemistry as a route to obtain a target specific lipid labeling [106]. Using an unnatural (BA modified) amino acid, it was able to preferentially label amine-presenting lipids via iminoboronate formation under physiological conditions. By targeting phosphatidylethanolamine and lysylphosphatidylglycerol, the two lipids enriched on bacterial cell surfaces, the iminoboronate chemistry allowed potent labeling of Gram-positive bacteria even in the presence of 10% serum, while bypassing mammalian cells and Gram-negative bacteria.

In the field of medicinal chemistry, a recent study by Su et al*.* have demonstrated that the iminoboronate chemistry can be exploited for the purpose of improved targeted covalent inhibition (TCI) for myeloid cell leukemia (Mcl-1), an anti-apoptotic protein [107]. The overexpression of Mcl-1 is a common mechanism for cancers to gain continuous resistance to apoptosis. Mcl-1 also represents a major target of protein-protein interaction (PPI), a critical regulation mode of cellular functions, and, therefore, the ability of inhibiting PPI would enable better understanding of key biological events and lead to the development of new molecular therapeutics [108–112]. A strict criterion for this type of application is that the covalent warheads used must not be merely highly reactive toward the target structure, as it may cause non-specific protein modification leading to toxicity, and yet the binding must be reversible. For this reason, modification of cysteines by weak electrophiles, such as electron-deficient olefins, is a standard strategy of TCI, although PPI targets do not always contain free cysteine residues in their protein-binding groove [113,114]. To design a reversible covalent inhibitor capable of targeting the ε-amino group of lysine, Su et al*.* incorporated a boronic acid carbonyl warhead into a previously reported indole-acid-based Mcl-1 inhibitor. The linker structure was first optimized in order to ideally situate the BA functionality in the vicinity of Lys234. On the basis of biochemical, cell-based as well as kinetic studies, these conjugates were proved to have dramatically enhanced and Mcl-1-specific potency as compared to noncovalent congeners.

## 3. Conclusions

This short review has highlighted recent approaches of BA chemistry-based biomaterials engineering with special focuses on its reversibly bio-interactive features. Generally weak and thus reversible molecular recognition of BA is a source of the richness in materials engineering, especially when combined with polymeric materials. Some emerging types of molecular targets including phosphates and nucleic acids may further draw interest to this area of the research. Meanwhile, research efforts pursuing material designs to deal with glycoconjugate class of structural complexity seems immature in quality with currently limited technical options, in which naturally occurring lectins are by far of magnificence. This may pose the ‘borono-lectin’ community an important challenge to better mimic the essence of lectins. With versatility in chemistry, inherent compatibility with life and growing accessibility, ‘borono-lectin’ continues to be an important platform for biomaterials science and engineering.

## Disclosure statement

No potential conflict of interest was reported by the authors.

## Funding

This work was supported by Grants-in-Aid for Scientific Research from the Ministry of Education, Culture, Sports, Science and Technology of Japan, Japan Science and Technology Agency [MEXT-JST: COI stream]; the Cooperative Research Project of Research Center for Biomedical Engineering [MEXT]; Japan Agency for Medical Research and Development [AMED: ACT-M program], Secom Science and Technology Foundation, Terumo Foundation for Life Sciences and Arts; Kanagawa Institute of Industrial Science and Technology (KISTEC-KAST).
